# Investigation on risk factors of haemorrhagic fever with renal syndrome (HFRS) in Xuancheng City in Anhui Province, Mainland China

**DOI:** 10.1017/S0950268820002344

**Published:** 2020-10-02

**Authors:** Guangjian Wu, Zhicai Xia, Fengtian Wang, Jiabing Wu, Deman Cheng, Xiaolong Chen, Huihui Liu, Zhongjun Du

**Affiliations:** 1 School of Public Health, Jilin University, Changchun, Jilin Province, People's Republic of China; 2 Shandong Center for Disease Control and Prevention, Jinan, Shandong Province, People's Republic of China; 3 Academy of Preventive Medicine, Shandong University, Jinan, Shandong Province, People's Republic of China; 4 Xuancheng Center for Disease Control and Prevention, Xuancheng, Anhui, Province, People's Republic of China; 5 Blood Centre for Shandong Province, Jinan, Shandong Province, People's Republic of China; 6 Anhui Center for Disease Control and Prevention, Hefei, Anhui Province, People's Republic of China; 7 Chinese Center for Disease Control and Prevention, Beijing, People's Republic of China; 8 Shandong Academy of Occupational Health and Occupational Medicine, Shandong First Medical University & Shandong Academy of Medical Sciences, Jinan, Shandong Province, People's Republic of China

**Keywords:** Haemorrhagic fever with renal syndrome (HFRS), Hantaviruses, Univariate analysis, Risk factors

## Abstract

Haemorrhagic fever with renal syndrome (HFRS), a rodent-borne disease, is a major public health concern in both developed and developing countries. China is the most severe endemic country in the world, constituting 90% of the cases. Although the incidence of HFRS has substantively decreased in most areas of China, HFRS has rebounded remarkably in some epidemic areas. Xuancheng is one of these areas. In this study, we collected the case data reported recently in Xuancheng and designed a 1:3 case−control study. The Chi-square test, univariate and multivariate logistic regression analysis were performed. In all cases, farmers made up the highest proportion of occupations. And there were 20 variables with statistical significance including indoor hygienic conditions; the surrounding environment; whether bitten by rats at work and other criteria. In addition, exposure to rodents and rats bites is a high-risk factor for HFRS. Rodent density was calculated at 20.9% (159/760), the virus carrier rate was 9.4% (15/159) and the index of rats with a virus was about 2.0%. Exposure to rodents and insect bites is also high-risk factors for HFRS among local residents in Xuancheng. More importantly, during the flood years, the increased density of rodents led to an increased risk of human exposure to rodents. As our statistical analysis proves, targeted strategies should be developed and implemented to reduce the incidence of local diseases in the future.

## Background

Haemorrhagic fever with renal syndrome (HFRS), also called epidemic haemorrhagic fever, is a rodent-borne disease caused by different species of hantavirus or Seoul virus and is characterised by fever, haemorrhage and acute renal dysfunction [1]. Asian and European continents are the major epidemic areas for HFRS [2], most of the documented HFRS cases annually occur in China, Korea and Russia [3]. Puumala virus is the most important hantavirus in Europe [4, 5] by contrast, Hantaan virus and Seoul virus are the two main serotypes of hantaviruses in China [6, 7]. China is the most severely endemic country in the world, with HFRS being prevalent for more than half a century, it has become a major public health problem and China accounts for over 90% of the total HFRS cases reported globally [8, 9]. Today, in mainland China, HFRS is endemic in each administrative province (autonomous regions and municipalities) and even in Hong Kong and Taiwan, human cases have been reported as well as infected animal hosts [10]. Since the year 2000, the incidence of HFRS has substantively decreased in most areas of China due to rodent control, vaccination, environmental management and other precautions. Nevertheless, HFRS has rebounded markedly in some epidemic areas, and has even been continuously present or re-emerged [11, 12].

Previous studies have suggested that the incidence of HFRS is influenced by socioeconomic situation (e.g. education level, primary occupation, resident income); climatic factors (e.g. density of the rodent population, prevalence of hantavirus infection in rodents); contact rate between rodents and humans and geological heterogeneity [6, 13, 14]. Although some scholars have tried to use models to find the connection between climate and host populations, however, it is difficult to distinguish the connection between climate and host populations in most places [1, 15].

Anhui Province, an administrative province in eastern China, is an epidemic area for HFRS. HFRS occurs in different degrees each year, peaking in spring and autumn. Annual average reported incidence of HFRS in Xuancheng has been among the top three in Anhui Province in recent years. Xuancheng is a famous tea-producing area. Most of the farmers are tea growers. Since the risk factors for the disease may have changed from previous reports, the aim here was to delineate the risk factors and to provide a basis for the prevention and control strategy for HFRS in Xuancheng.

## Materials and methods

The case data were collected from Compilation of Epidemic Data in Xuancheng and China Information System for Disease Control and Prevention. Population data come from *Xuancheng Statistical Yearbook*. The principle of control selection is that the age and gender is the same as the case in the same village (the same community), and people who are not sick are taken as the control.

### Investigation of risk factors

We collected information about a month before the onset of the case (the ‘pre-morbidity’ in the control exposure history refers to the onset time of the case paired with it). The information includes general information (e.g. name, gender, age, primary occupation, phone number); incidence (e.g. onset time, place of onset, visiting time, diagnosed time); living environment (e.g. housing type, indoor hygiene; indoor rodent holes, indoor rodent population; surrounding environment; housing location; whether the housing was adjacent to ponds or rivers whether there were firewood piles around the housing; type of living floor; whether there were rodents in the workplace); whether wearing protective clothing at work; having contact with rodents and so on. Overall there were more than 30 criteria employed.

### Survey on virus-carrying status of rats

We selected Xuanzhou District, Guangde County and Langxi County as survey areas with high incidence. Each survey area selected three high incidence sites and two villages were selected for each site. The principles for selecting survey sites and villages are as follows: Towns (villages) with persistent case reports from 2015 to 2016, if the number of towns (villages) reported from 2015 to 2016 was insufficient, then the towns (villages) with the largest number of cases reported in 2014 and no further cases reported after 2014 were selected. In rural residential areas, representative natural villages with HFRS cases were selected. Survey of rodent density in the field was investigated within the radius of 500 m outside the selected natural village.

We chose places where rodents might live, such as rivers, canals, roads, ridges, graveyards and courtyards. We used the nip capturing method to catch rats, with traps placed in selected locations in the evening followed by collection next morning. In residential areas, 1 trap per 10 m^2^ and 2 rat traps larger than 10 m^2^ were used and five traps were placed in an area for 30 houses. Traps were placed in the place where rodents often hunt in the evening and collected next morning. In field areas, we chose forestry and farmland to conduct the investigation of rodent density, with 1 trap every 5 m and a 50 m row spacing. Traps were placed in the place in the evening and collected next morning. The bait constituted of peanuts and the types of rodents captured were recorded. We dissected the classified and identified rodents aseptically, rodent lungs were screened for pathogenesis. Rodent density was calculated as a proportion (total number of captured rodents/total number of valid traps). An invalid rat trap was defined as either a missing trap or non-rodent triggered trap. The virus carrier rate was also calculated as a proportion (number of rodents with the virus/total number of captured rodents).

### Data collation and analysis

Using Epidata 3.1 to double input data, chi-square test was used for trend analysis of counting data. The risk factors of exposure were analysed by univariate analysis. Multivariate logistic regression analysis was performed for the statistically significant exposure factors. Chi-square test, univariate and multivariate logistic regression analysis were performed with SPSS18.0 software, *P* < 0.05.

## Results

### Investigation of risk factors

Newly reported cases of HFRS in Xuancheng in 2016 were selected as the case group and case inclusion was up to the end of November 2016. According to the same village (same community), age and sex-matched individuals without evidence of disease were chosen as the control group, and the control group was selected according to 1:3. A total of 42 paired cases and controls were investigated by questionnaires. The median age of cases and controls was 48 and 49, respectively.

### Time, regional and population distribution characteristics

**Fig. 1.**
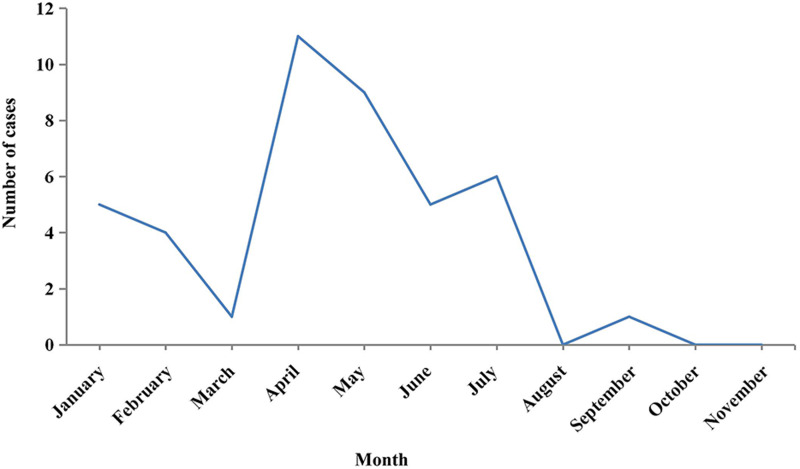
Monthly report of HFRS cases in three regions of Xuancheng from January to November, 2016.

Figure 1 shows that of the 42 collected cases, these were reported mainly in April and May. Among the three regions where cases were collected, Langxi County had the largest number of reported cases, accounting for half of the total reported cases. Xuanzhou District had the lowest number of cases, accounting for only 21.4% as can be seen in Table 1. Of the 42 cases, farmers accounted for the highest proportion (66.7%), followed by workers (9.5%). In all, 89.3% (25/28) of farmers were tea growers, as shown in Table 2.

**Table 1.** Region distribution of reported HFRS cases in three regions of Xuancheng from January to November, 2016

Region	Number of cases	%
Langxi county	21	50.0
Guangde county	12	28.6
Xuanzhou district	9	21.4
Total	42	100.0

**Table 2.** Occupation distribution of reported HFRS cases in three regions of Xuancheng from January to November, 2016

Occupation	Number of cases	%
Farmer	28	66.7
Worker	4	9.5
Student	2	4.8
Business service personnel	2	4.8
Migrant worker	2	4.8
Others	2	4.8
Fishermen (boatmen)	1	2.4
Retired personnel	1	2.4

### Univariate analysis of risk factors

There were 20 variables with statistical significance (*P* < 0.05), including housing type, indoor hygiene conditions, having rat holes in the room, number of indoor rats, surrounding environment, type of living floor, tea grower or not, whether there were rats in the workplace, whether they wore protective clothing at work, being bitten by rats at work, contact with water while sweeping the floor in the room (or courtyard), maintaining dogs or pigs at home, exposure to rodents or having eaten food contaminated by rodent excreta. Other criteria included having contact with an HFRS confirmed case, having direct contact with excreta of poultry or livestock, sitting or lying near a rat hole, having being accommodated in the field or having a history of insect bites. Among all of these criteria, having good indoor hygienic conditions, wearing protective appliances at work or being in contact with water while sweeping the floor in the room (or courtyard) were protective factors against the disease, as shown in Table 3.

**Table 3.** Univariate analysis of the 1: 3 case−control study of HFRS in Xuancheng

Variable	*χ*^2^	*p*	OR (95% CI)
Housing type (bungalow, a building of two or more storeys)	13.34	0.00	4.01 (1.85−8.70)
Indoor hygiene condition (good, general or bad)	38.23	0.00	0.10 (0.04−0.22)
Having rat holes in the room (yes or no)	54.88	0.00	51.22 (11.16−234.99)
Number of indoor rats (more, less or none)	37.51	0.00	56.03 (7.04−445.72)
Surrounding environment (having grain or canteen or warehouse or no)	13.34	0.00	4.01 (1.85−8.70)
Housing location (in, by or outside the village)	0.20	0.65	1.18 (0.58−2.38)
Housing adjacent to ponds or streams (yes or no)	1.19	0.28	1.48 (0.73−2.99)
Having firewood piles around the housing (yes or no)	0.04	0.85	1.07 (0.52−2.23)
Type of living floor (clay, bricks or cement)	8.83	0.00	2.94 (1.42−6.07)
Tea grower (yes or no)	7.35	0.01	2.65 (1.29−5.42)
There are rats in the workplace (yes or no)	17.84	0.00	4.83 (2.25−10.36)
Wearing protective appliances at work (yes or no)	21.66	0.00	0.13 (0.05−0.34)
Bitten by rats at work (yes or no)	63.64	0.00	–
Watering while sweeping the floor in the room or courtyard (yes or no)	13.71	0.00	0.26 (0.12−0.54)
Maintain dogs at home (yes or no)	7.89	0.00	2.79 (1.34−5.77)
Maintain pigs at home (yes or no)	10.90	0.00	4.61 (1.76−12.12)
Maintain cats at home (yes or no)	0.37	0.54	1.41 (0.46−4.33)
HFRS cases have been reported in villages or in families (yes or no)	1.07	0.30	1.74 (0.60−5.04)
Exposure to rodents (yes, no or unknown)	90.95	0.00	124.00 (26.65−576.87)
Have eaten food contaminated by rodent excreta (yes, no or unknown)	−	0.02	–
Having contacted with HFRS confirmed case (yes, no or unknown)	31.16	0.00	–
Having direct contact with excreta of poultry or livestock (yes, no or unknown)	11.61	0.00	–
Once sitting or lying near a rat hole (yes, no or unknown)	11.61	0.00	–
Once sitting or lying on firewood in the yard (yes, no or unknown)	–	0.06	–
Once accommodation in the field (yes, no or unknown)	–	0.02	–
Having a history of insect bites (yes, no or unknown)	11.18	0.00	20.83（2.43−178.72）

Note: ‘-’ represents uncalculable.

### Multivariate logistic regression analysis of risk factors

Of the 20 variables selected by univariate analysis, there were five variables for which there was no evidence of exposure in the control group. These included being bitten by rats at work; having eaten food contaminated by rodent excreta; having being in contact with an HFRS confirmed case; being in direct contact with excreta of poultry or livestock; sitting or lying near a rat hole or having taken accommodation in the field. If the five variables were included in the multivariate analysis, the impact of other variables on the outcomes would be concealed, so they were excluded from the multivariate logistic regression analysis. Multivariate logistic regression analysis was carried out for the remaining 15 variables screened out from univariate analysis. Variables were screened by ENTER method. The criteria for entering the equation was *P* < 0.05, and for eliminating the equation was *P* > 0.10. Three significant factors were identified, including surrounding environment, exposure to rodents and having a history of insect bites, as shown in Table 4.

**Table 4.** Multivariate logistic regression analysis of the 1:3 case−control study of HFRS in Xuanchen

Variable	*β*	SE (*β*)	Wald	*p*	OR	95% CI
Surrounding environment	2.07	0.89	5.42	0.02	7.909	1.386−45.136
Exposure to rodents	4.61	1.09	17.83	0.00	99.951	11.789−847.403
Having a history of insect bites	3.56	1.39	6.51	0.01	34.998	2.281−536.862

### Survey on virus-carrying status of rats

In a total of 760 valid traps, 159 rats were captured, including 137 *Apodemus agrarius*, 13 sewer rats, 8 *Mus musculus* and 1 *Rattus flavipectus*. Rodent density was calculated as 20.9% (159/760), and the virus carrier rate was 9.4% (15/159). The index of rats with virus was about 2.0% (20.9% × 9.4%).

## Discussion

Previous research studies have provided evidence that habitat environment, climate and other geo-ecological conditions affect the composition and density of the host animal population, which determines the type and intensity of hantavirus epidemic areas [16]. Our study shows that *Apodemus agrarius* and sewer rats were the major types of rodents captured in Xuancheng. *Apodemus agrarius* is distributed almost entirely across the Asian and European continents and it can carry many different subtypes of Hantaan virus [17]. In China, *Apodemus agrarius* prefers to live in agricultural areas, grasslands near water, forest edges and logging lands, and may even enter forest residential areas for temporary or long-term residence. That means people have a high probability of exposure to *Apodemus agrarius* when they are engaged in agricultural production activities. Our analysis supports the above view. In this study, farmers especially tea growers accounted for the highest proportion of reported cases. Langxi and Guangde counties are well recognised as tea-producing hilly areas. Spring is the busiest season for tea growing and picking. Tea growers work in hilly areas without protective clothing, increasing the risk of contact with rats and even being bitten by them, and also increasing the chance of being bitten by insects in the field. Our univariate analysis also suggests that direct or indirect exposure to rodents and their excreta is a risk factor for HFRS. Multivariate analysis showed that exposure to rodents and insect bites are high-risk factors for HFRS.

The sewer rat is the main host of the Seoul virus, a moist tropical rodent. The sewer rat has spread all over the world with the development of the shipping industry, adjusting itself to the human living environment. Current studies have found that Seoul virus RNA nucleotide composition differences are relatively small throughout the world [18]. This means that the current prevalence of HFRS in the world is not mainly caused by Seoul virus. In China, Hantaan virus is responsible for up to 70% of cases [19].

According to Trophic cascade, in flood years, rainwater is abundant, crops grow vigorously and rat food is abundant, which is conducive to rat reproduction. When the rat density reaches a certain level, the transmission mode of hantavirus will change, resulting in the acceleration of rat infection [20]. Our research indirectly proves this view since severe flooding occurred in the provinces of the Yangtze River Basin in 2016, one of which is Anhui Province. The rivers in Xuancheng mainly belong to the Yangtze River basin system, and rodent density obtained in this study was found to be much higher than that obtained in previous surveillance.

Based on our investigation results, we suggest that farmers especially tea growers should wear protective clothing, avoiding direct or indirect exposure to rodents and their excreta and insect bites when working in hilly areas. If necessary, deratisation should be carried out to reduce the density of rats.

## Conclusions

Exposure to rodents and insect bites are high-risk factors for HFRS among local residents in Xuancheng. In addition, in flood years, the abundance of rainwater and food leads to an increase in rodent density, resulting in increased exposure to rodents by humans. As evidenced in our statistical analyses, targeted strategies should be formulated and implemented to reduce future local disease incidence.

## Limitations of this study

This study has the following limitations: First, previous studies suggested that climate is an important factor affecting HFRS occurrence, but this study was constrained that did not include climate factors in the analysis which is one of the number of major limitations. Second, being constrained by the number of samples surveyed, five meaningful variables of no exposure in the control group were not included in the multivariate analysis, which did not allow us to further confirm the role of these factors. These limitations may be addressed in better design future studies.

## Declarations

### Ethics approval and consent to participate

Ethics and approval were sought and granted from Shandong Center for Disease Control and Prevention (Approval for ethical review of biomedical research involving humans, ETH-2017020), and obtained the written informed consent of all the participants. Participation of subjects was voluntary and written consent was obtained from all participants prior to data collection.

### Availability of data and materials

All data and materials for this study shall be availed whenever requested by editorial team, reviewers and other users. The data set can be accessed by sending a request to duzj1981@163.com.
